# The Time-Dependent Effects of Temozolomide on Autophagy Gene Expression in Glioblastoma Cells

**DOI:** 10.3390/biomedicines14030656

**Published:** 2026-03-13

**Authors:** İlker Kiraz, Veli Kaan Aydın, Özgür Kurt, Mehmet Erdal Coşkun, Gergana Lengerova, Martina Bozhkova, Steliyan Petrov, Aylin Köseler

**Affiliations:** 1 Department of Neurosurgery, Faculty of Medicine, Pamukkale University, Denizli 20160, Turkey; ikiraz@pau.edu.tr (İ.K.); mecoskun@pau.edu.tr (M.E.C.); 2 Department of Biophysics, Faculty of Medicine, Pamukkale University, Denizli 20160, Turkey; vkaydin@pau.edu.tr; 3 Department of Medical Microbiology, Acibadem Mehmet Ali Aydinlar University School of Medicine, Istanbul 34752, Turkey; ozgur.kurt@acibadem.edu.tr; 4 Department of Medical Microbiology and Immunology “Prof. Dr. Elissay Yanev”, Medical University of Plovdiv, 4002 Plovdiv, Bulgaria; gergana.lengerova@mu-plovdiv.bg (G.L.); martina.bozhkova@mu-plovdiv.bg (M.B.); steliyan.petrov@mu-plovdiv.bg (S.P.); 5 Research Institute, Medical University of Plovdiv, 4002 Plovdiv, Bulgaria

**Keywords:** glioblastoma, temozolomide, autophagy, time-dependent gene expression, chemoresistance

## Abstract

**Background**: Temozolomide (TMZ) resistance represents a major therapeutic challenge in glioblastoma treatment, where autophagy has emerged as a key adaptive survival mechanism. Although numerous studies have implicated autophagy in TMZ resistance, most have assessed this process at a single point, thereby overlooking its dynamic and time-dependent nature. **Methods**: In this study, we systematically investigated the temporal regulation of autophagy-related gene expression in two human glioblastoma cell lines with distinct MGMT methylation status and TMZ sensitivities (T98G and U87) following TMZ treatment. Cells were exposed to TMZ and harvested at defined time points (0 h, 6 h, 24 h, and 48 h). The expression levels of genes representing distinct stages of the autophagy pathway, including initiation, nucleation, elongation, selective autophagy, lysosomal function, and transcriptional regulation, were analyzed using RT-qPCR. Relative gene expression was calculated using the 2^−ΔΔCT^ method with GAPDH as the reference gene. **Results**: Our results reveal a time-dependent and phase-specific transcriptional reprogramming of the autophagy machinery in response to TMZ-induced stress. Early time points were characterized by modulation of autophagy initiation and nucleation genes, whereas intermediate and late phases showed prominent regulation of genes associated with autophagosome elongation, selective autophagy, autophagic flux, and transcriptional control. **Conclusions**: Collectively, these findings demonstrate that autophagy in TMZ-treated glioblastoma cells is not a static response but a dynamically regulated, multi-phase program. Specifically, in TMZ-resistant T98G cells, this process matures into a sustained adaptive program with robust late-phase lysosomal integration, while in TMZ-sensitive U87 cells, the early autophagic response is transient and fails to support long-term lysosomal coordination. This temporal perspective provides new insights into the role of autophagy in TMZ tolerance and underscores the importance of time-resolved analyses when targeting autophagy to overcome chemoresistance in glioblastoma.

## 1. Introduction

Glioblastoma multiforme is the most frequent type of brain tumor in adults and also the most malignant type. Unfortunately, due to the limitations of current treatment methods, the average survival time for patients continues to be in a very narrow range of 14–15 months [1]. This situation is associated with the aggressive nature of the tumor and the resistance mechanisms developed against the treatments [2]. Contrary to the fact that Temozolomide is being widely used as a chemotherapy agent in glioblastoma treatments, it is still able to cause resistance in about 50% of cases, thus significantly decreasing the success of the treatment [2]. Therefore, understanding and overcoming temozolomide resistance are essential keys to survival rate increases in glioblastoma treatment [3,4]. One of the major mechanisms of temozolomide resistance in glioblastoma cells is the increase in the capacity of the tumor cells to survive through drug-induced autophagy [5]. Autophagy is a catabolic process whereby cells digest and recycle their own components, and it has been demonstrated to play an important role in the development of resistance against temozolomide in glioblastoma [4]. This mechanism allows cancer cells to survive under stress conditions; however, it has been suggested that excessive autophagy can lead to cancer cell death [4,6]. This contradictory role reveals the complexity and the potential of targeting autophagy in anticancer therapies [4]. Therefore, studying the time-dependent effects of temozolomide on autophagy gene expression in glioblastoma cells is of great importance for the development of therapeutic strategies [7]. The clarification of these mechanisms will be a basis for overcoming temozolomide resistance and developing more effective therapeutic approaches for glioblastoma patients [2,4]. Moreover, it has been suggested that blocking autophagy may increase the temozolomide sensitivity of glioblastoma stem cells [8]. In this regard, a detailed analysis of the time-dependent effects of temozolomide on autophagy-related genes in glioblastoma cells will contribute significantly to the molecular understanding of resistance mechanisms [2,9,10].

Glioblastoma multiforme is the most aggressive form of astrocytoma and is classified as a WHO Class IV malignancy; it also has the highest incidence among central nervous system tumors [11]. It has an annual incidence rate of 5.26 per 100,000 people; around 17,000 new cases are diagnosed annually, accounting for almost 80% of primary brain tumors [12]. In children, glioblastoma accounts for about one-fifth of all childhood cancers. The high aggressiveness of glioblastoma is due to its characteristic features of rapid cell proliferation, invasive growth, and angiogenesis, while resistance to current treatment protocols makes the prognosis even worse [13]. The heterogeneous nature of these tumors, with different genetic and molecular subtypes, adds to their complexity, thus necessitating the development of personalized therapeutic approaches [14]. In line with this, the molecular-level understanding of glioblastoma pathophysiology is crucial for the development of effective therapeutic strategies. Because of the high recurrence rates of the disease and the resistance which develops against the therapy, identifying molecular targets and discovering new drug candidates require extensive research [15]. Furthermore, delineating cellular processes such as autophagy in glioblastoma can reveal novel therapeutic targets that potentially improve survival [16,17].

Temozolomide is accepted as a standard oral alkylating agent in the treatment of glioblastoma multiforme, and it hinders the growth of tumor cells by alkylation of DNA [18]. However, resistance to temozolomide occurs in about 50% of patients, which is an important factor limiting the effectiveness of the drug [16]. This resistance has been linked to mechanisms such as high expression of the O6-methylguanine-DNA methyltransferase gene, as this enzyme removes the alkylating effect of temozolomide on DNA and thus provides protection against chemotherapy [4]. Therefore, strategies such as MGMT inhibitors are being developed to overcome temozolomide resistance, and new treatment approaches targeting these resistance mechanisms are being explored. However, it is possible that the inadequacy of temozolomide monotherapy due to its side effects such as myelotoxicosis, nausea, and fatigue may pave the way for tumor recurrence [3]. These limitations reveal the need for alternative or combination therapies to increase the efficacy of temozolomide and to overcome resistance mechanisms in glioblastoma treatment [3]. In this regard, a thorough investigation of the molecular pathways that constitute the basis of temozolomide resistance, especially autophagy and other cellular defense mechanisms, can provide novel therapeutic targets to overcome treatment failure [5,19].

Autophagy is a vital catabolic process through which cells recycle components by degrading their damaged or dysfunctional organelles and proteins via lysosomes. This mechanism is critically important to maintain cellular homeostasis, respond to stress, and combat a variety of pathological conditions [20]. In the context of cancer, autophagy can function as a tumor suppressor during the early stages of tumorigenesis, but at later stages, it helps tumors survive chemotherapy and radiotherapy by developing resistance [21,22]. This dual role has led to the consideration of autophagy in cancer therapy as both a potential target and a resistance mechanism [5]. Therefore, the timing and extent of autophagy activation may be crucial factors determining the success of cancer treatment. It has been shown, in particular, that autophagy acts as a cytoprotective mechanism in the development of temozolomide resistance and thus cancer cells acquire greater survival ability under TMZ treatment [21,23]. In this context, increased autophagic flux was detected in temozolomide-resistant glioblastoma cells, and it was associated with a decrease in the levels of autophagy markers LC3A/B-II and p62 [24]. Here, it is suggested that cytoprotective autophagy contributes to the resistance of glioblastoma cells to chemotherapy by inhibiting TMZ-induced apoptosis [25,26]. Hence, the complicated and paradoxical role of autophagy in cancer is reinforced by findings that it can both promote and suppress tumor growth [27]. Given that TMZ sensitivity is not consistent between and within individual malignant glioma cell lines due to divergent genetic backgrounds, such as varying MGMT methylation status, relying on a single cell line limits the generalizability of the findings. Therefore, to overcome this limitation and better understand the dynamic role of autophagy, we comparatively evaluated these time-dependent effects in both T98G (MGMT-unmethylated, TMZ-resistant) and U87 (MGMT-methylated, TMZ-sensitive) cell lines.

## 2. Materials and Methods

### 2.1. Cell Line and Culture Conditions

Human glioblastoma cell lines T98G (ATCC: CRL-1690) and U87-MG (ATCC: HTB-14) were obtained from the American Type Culture Collection (ATCC, Manassas, VA, USA). Both cell lines were cultured in Dulbecco’s Modified Eagle Medium (DMEM; high glucose; Gibco, Billings, MT, USA) supplemented with 10% fetal bovine serum (FBS; Capricorn Scientific, Ebsdorfergrund, Germany) and 1% penicillin/streptomycin (Gibco, Billings, MT, USA). Cells in the logarithmic growth phase were maintained at 37 °C in a humidified incubator with 5% CO_2_ and were passaged two to three times per week. All experimental procedures were conducted under identical culture conditions for both cell lines.

### 2.2. Preparation for Temozolomide Doses

A stock solution of temozolomide (TMZ; TEMODAL®, Merck Sharp & Dohme, Rahway, NJ, USA) was prepared by dissolving the compound in 100% dimethyl sulfoxide (DMSO; Thermo Fisher Scientific, Waltham, MA, USA). Due to the light sensitivity of TMZ, stock solutions were prepared in amber tubes, aliquoted, and stored at −20 °C. A fresh aliquot was used for each experimental day. The stock solution was serially diluted in fresh culture medium to obtain final concentrations of 50 µM, 100 µM, and 200 µM. The final DMSO concentration was kept constant across all treatment groups and matched with the vehicle control (DMSO only). Both T98G and U87 cells were treated under identical dosing conditions.

### 2.3. MTS Cell Viability Assay

Cell viability was assessed using the MTS [3-(4,5-dimethylthiazol-2-yl)-5-(3-carboxymethoxyphenyl)-2-(4-sulfophenyl)-2H-tetrazolium] (Sigma Chemical Co., St. Louis, MO, USA) assay. T98G and U87 cells were seeded at a density of 5 × 10^3^ cells per well in 96-well plates and allowed to attach for 24 h prior to treatment. Cells were then exposed to TMZ (50, 100, and 200 µM) for 6 h, 24 h, and 48 h. At the end of each treatment period, MTS reagent was added according to the manufacturer’s instructions and absorbance was measured at 450 nm using a Multiskan FC Microplate Photometer (Thermo Fisher Scientific, Waltham, MA, USA). All experiments were performed in triplicate. Cell viability was calculated as a percentage relative to the corresponding vehicle-treated control for each cell line.

### 2.4. RNA Isolation

Total RNA was isolated from the cells at the designated time points, using RNeasy Mini Kit (Qiagen, Hilden, Germany) according to the manufacturer’s protocol. The amount and purity of RNA were evaluated by spectrophotometric measurement (NanoDrop 2000, Thermo Fisher Scientific, Waltham, MA, USA), and only the samples that met the quality criteria were used for the analyses.

### 2.5. Complementary DNA (cDNA) Synthesis

cDNA synthesis from Total RNA was performed according to the protocol of the RevertAid First Strand cDNA Synthesis Kit (Thermo Fisher Scientific, Waltham, MA, USA). Same amount of total RNA (1 µg) was used for each sample. Reverse transcription reactions were run by using Veriti^TM^ 96-Well Thermal Cycler (Applied Biosystems, Foster City, CA, USA). The samples were incubated at 25 °C for 5 min, 42 °C for 60 min, and followed by incubation at 70 °C for 5 min to stop the reaction.

### 2.6. RT-qPCR Analysis of Autophagy Gene Expression

The expression levels of the genes that represent the different phases of the autophagy process were analyzed by the RT-qPCR method using a CFX96 Real-Time PCR System (Bio-Rad Laboratories, Hercules, CA, USA). All samples were run as technical triplicates and amplification specificity was confirmed by melting curve analysis. The autophagy-related genes analyzed in this study included *ULK1*, *BECN1* (*Beclin-1*), *ATG5*, *ATG7*, *WIPI1*, *LC3B*, *SQSTM1* (*p62*), *NBR1*, *FOXO3*, and *TFEB*, representing key regulatory steps of the autophagy pathway, including initiation, elongation, selective autophagy, and transcriptional regulation. Primer sequences used for RT-qPCR analysis of these genes are listed in Table 1. All primer sequences are presented in the 5′–3′ direction, and primer specificity was verified by melting curve analysis. *GAPDH* was used as the reference (housekeeping) gene for normalization of gene expression.

### 2.7. Gene Expression Data Analysis

The CT values obtained from RT-qPCR analyses were first normalized to each sample by subtracting CT values of the reference gene GAPDH from the target genes (ΔCT). Then, ΔCT values at each time point were compared to the mean ΔCT value of the control group (0 h, vehicle-treated) to calculate ΔΔCT values. Relative gene expression levels were determined using the 2^−ΔΔCT^ method. The average CT values were taken over technical replicates. The results were expressed as fold changes relative to the control group. Time-dependent changes were assessed by comparing the relevant time points with the control group. The results were reported as mean ± standard error of the mean (SEM).

### 2.8. Statistical Analysis

All experiments were performed in at least three independent biological replicates. Data are presented as mean ± standard error of the mean (SEM). Statistical analyses were conducted using one-way analysis of variance (ANOVA) followed by suitable post hoc tests. A *p* value < 0.05 was considered statistically significant (with using GraphPad Prism version 9.0, GraphPad Software, Boston, MA, USA).

## 3. Results

Based on dose-range assessments, TMZ was evaluated at final concentrations of 50 µM, 100 µM, and 200 µM to determine an appropriate exposure level for time-course analyses. Cell viability in the control group (medium only) was 100%. These experiments indicated that 100 µM TMZ elicited robust cellular responses without inducing excessive acute cytotoxicity (Table 2).

Accordingly, 100 µM TMZ was selected as the final experimental concentration for all subsequent time-dependent gene expression analyses. To investigate the time-dependent regulation of autophagy-related gene expression, cells treated with 100 µM TMZ were harvested at predefined time points: 0 h, 6 h, 24 h, and 48 h following drug exposure.

**Figure 1:**
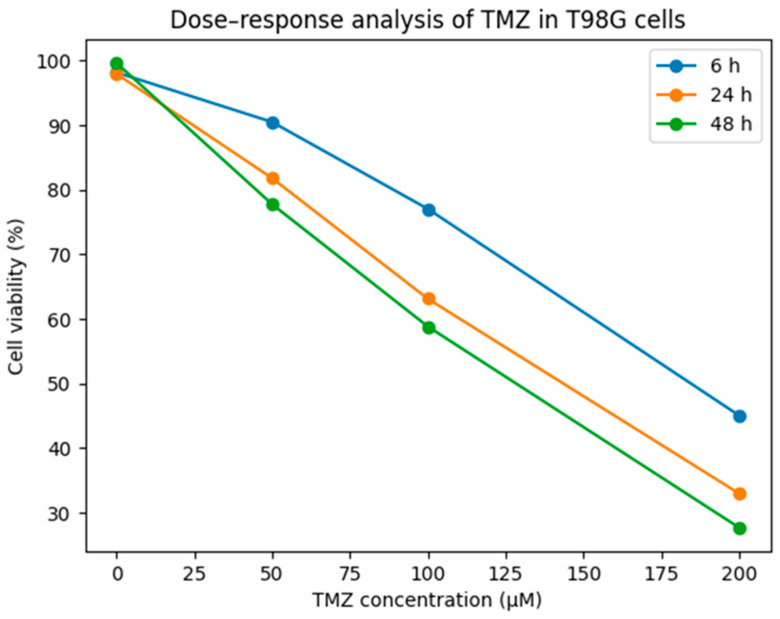
Dose–response analysis of temozolomide (TMZ) in T98G glioblastoma cells.

T98G cells were exposed to 50, 100, and 200 µM TMZ. Cell viability was measured at 6 h, 24 h, and 48 h time points and vehicle (DMSO) was normalized. The data are presented in Figure 1 as the mean of three independent experiments. TMZ has caused a reduction in cell viability in a time- and dose-dependent manner.

Cell viability was assessed at 6, 24, and 48 h at TMZ concentrations of 0, 50, 100, and 200 µM; values are expressed as mean viability (%).

**Figure 2:**
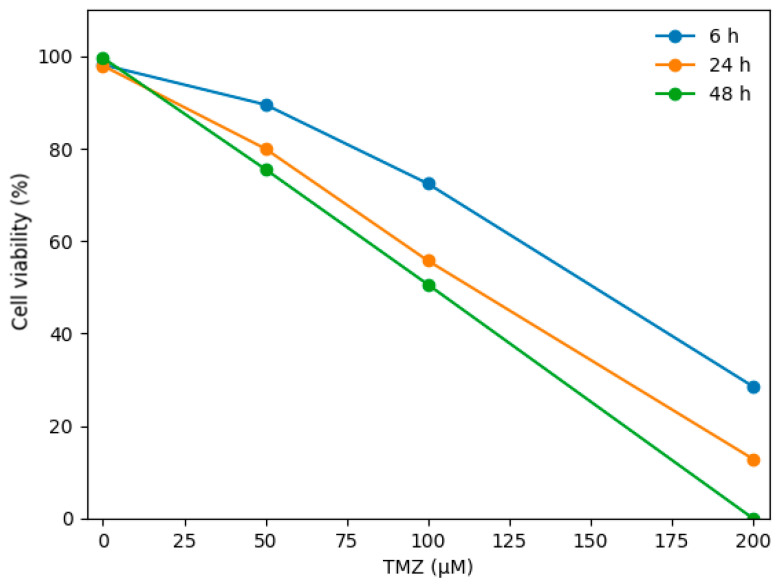
Dose–response analysis of temozolomide (TMZ) administration in U87 glioblastoma cells.

**Figure 3:**
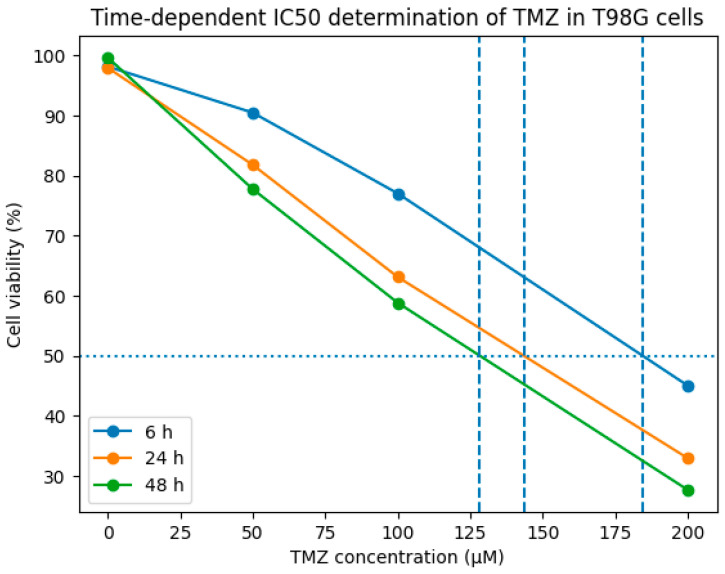
Time-dependent determination of IC50 values for temozolomide (TMZ) in T98G cells.

Dose-, response-, and time-dependent cell viability assays were performed to determine the most appropriate TMZ concentration for subsequent gene expression analyses in both T98G and U87 glioblastoma cells (Figure 1 and Figure 2). In both cell lines, 100 µM TMZ induced a gradual reduction in cell viability over time. However, this concentration was selected as a sublethal dose for RT-qPCR analyses, as it did not cause extensive cell death, particularly at early time points, thereby allowing reliable assessment of time-dependent transcriptional changes. The cytotoxic effect of temozolomide (TMZ) in T98G glioblastoma cells and the corresponding IC50 values were determined from dose–response curves (Figure 3). Cell viability was measured at time points of 6 h, 24 h, and 48 h. The horizontal dashed line indicates 50% cell viability, and the vertical dashed lines represent the IC50 values calculated at each time point. The IC50 values were determined to be approximately 185 µM for 6 h, 144 µM for 24 h, and 128 µM for 48 h, respectively.

The IC50 values calculated based on dose–response analyses showed that TMZ exhibited a time-dependent cytotoxic effect in T98G cells. The IC50 values were determined to be approximately 185 µM for 6 h, 144 µM for 24 h, and 128 µM for 48 h, respectively.

Cell viability was assessed following exposure to increasing concentrations of TMZ (0–200 µM) at 6 h, 24 h, and 48 h. Dose–response curves were generated using a four-parameter logistic regression model. The horizontal dashed line indicates 50% cell viability, and the vertical dashed lines represent the corresponding IC_50_ values at each time point (6 h: 146.37 µM; 24 h: 107.28 µM; 48 h: 94.61 µM).

**Figure 4:**
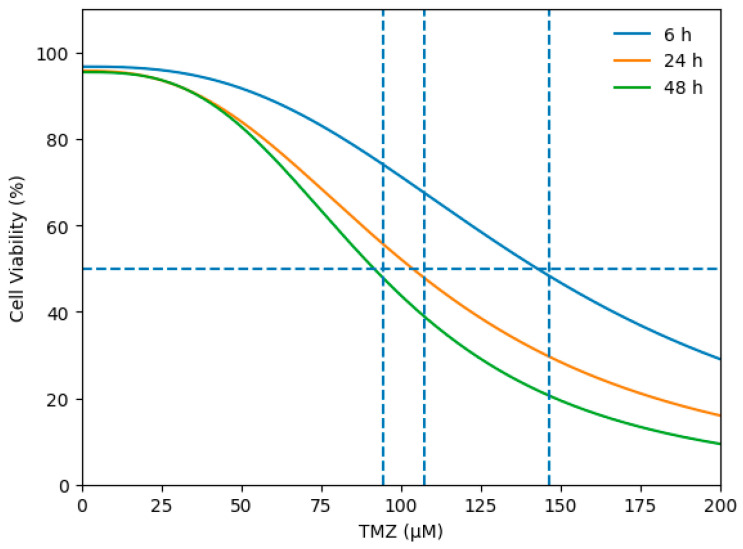
Time-dependent determination of IC_50_ values of temozolomide (TMZ) in U87 glioblastoma cells.

The cytotoxic effect of temozolomide (TMZ) in U87 glioblastoma cells and the corresponding IC50 values were determined from dose–response curves (Figure 4). Cell viability was evaluated at 6 h, 24 h, and 48 h. The horizontal dashed line represents 50% cell viability, while the vertical dashed lines indicate the calculated IC50 values at each time point. The IC50 values were calculated as 146.37 µM at 6 h, 107.28 µM at 24 h, and 94.61 µM at 48 h, demonstrating a progressive, time-dependent increase in TMZ sensitivity in U87 cells.

**Figure 5:**
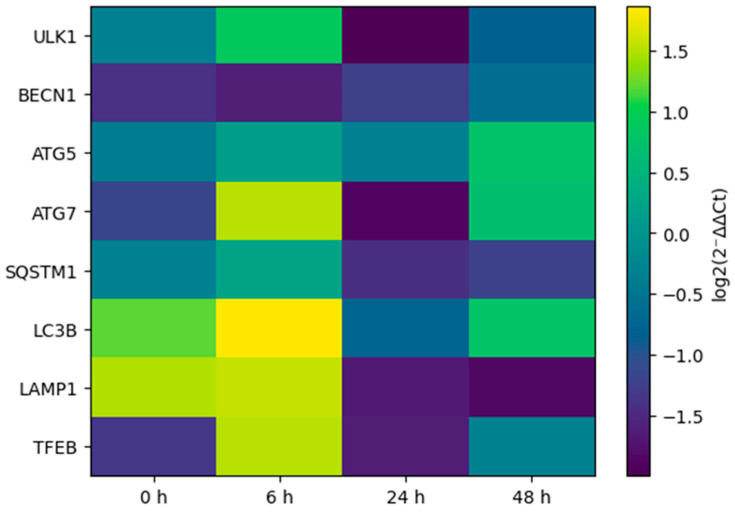
Time-dependent autophagy gene regulation under temozolomide.

In order to evaluate the time-dependent effects of TMZ treatment on the expression of autophagy-related genes, RT-qPCR analyses were performed on T98G glioblastoma cells at different time points. The results obtained showed that the response of autophagy genes to TMZ was time-dependent. Most of the genes analyzed displayed different expression patterns at the early, middle and late phases after TMZ treatment (Figure 5). The time-normalized gene expression profiles reveal that the autophagic response is not a unidirectional increase or decrease but a dynamically reprogrammed process.

Heatmap representation of time-dependent changes in autophagy-related gene expression in T98G glioblastoma cells following temozolomide treatment. Relative expression values were calculated using the 2^−ΔΔCT^ method normalized to *GAPDH* and are displayed as log2(2^−ΔΔCT^) relative to the 0 h vehicle control. Color intensity reflects the magnitude and direction of regulation over time. No z-score standardization was applied. Genes involved in autophagy initiation, elongation, selective autophagy, lysosomal function, and transcriptional regulation are shown. Color intensity represents the magnitude of gene regulation over time, highlighting dynamic and phase-specific transcriptional reprogramming of the autophagy machinery.

**Figure 6:**
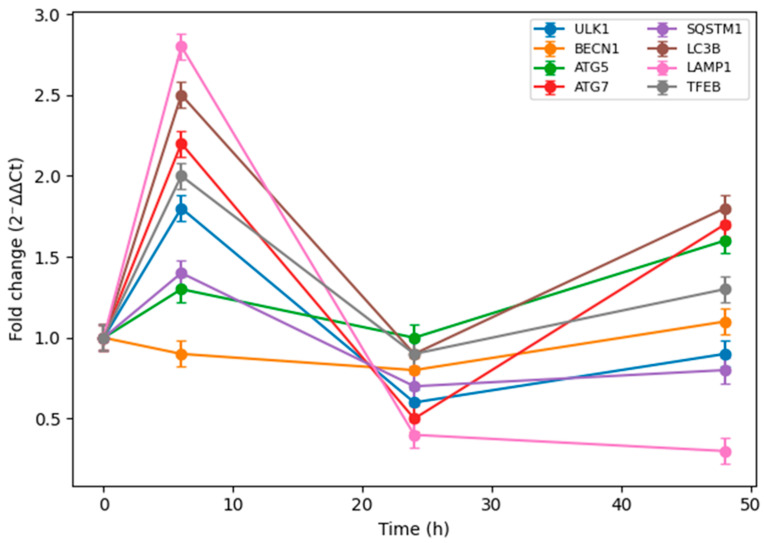
Phase-specific temporal modulation of autophagy-related genes in response to temozolomide.

After the application of TMZ, marked changes were observed at the early time point (6 h) in the expression levels of the genes involved in the initiation and nucleation phases of autophagy (Figure 6). More specifically, a significant increase in *ULK1* gene expression, which plays a crucial role in the initiation of autophagy, at 6 h strongly suggests that the mechanisms of autophagy initiation were activated at the early stage as a rapid stress response to TMZ.

On the other hand, it was noted that during the nucleation process of autophagy, the expression level of the *BECN1* gene, which is one of the main regulators of this process, did not show a significant increase at the early phase, and up to 24 h it exhibited a limited or repressed expression profile. This finding reflects that during the early phase of autophagy in response to TMZ, the *ULK1*-mediated initiation signal predominates, while the nucleation step might be temporally delayed or a more tightly regulated step of the process.

At a later time point (48 h), it was observed that both *ULK1* and *BECN1* gene expression levels were close to basal values or slightly increased, indicating that the steps of autophagy initiation and nucleation after the early phase have been rebalanced and integrated into the cellular adaptation process.

RT-qPCR analysis shows time-dependent changes in the expression of autophagy-related genes in T98G cells treated with temozolomide. Relative gene expression levels were calculated using 2^−ΔΔCT^ method normalized to *GAPDH* and are presented as fold change relative to the 0 h vehicle control. Data represent mean ± SEM of at least three independent experiments.

Following TMZ treatment, it was observed that the expression profiles of the genes involved in the later stages of autophagy became particularly apparent at a late time point (48 h). The increased expression levels of *ATG5* and *ATG7* genes at 48 h, which are involved in the autophagosome elongation, show that the late phases of autophagy are actively turned on in T98G cells exposed to TMZ. This increase might indicate that the autophagic response initiated at the early phase is maturing over time and gradually moving to the elongation and autophagosome formation steps.

In parallel, the regulation of *SQSTM1* (*p62*) gene expression, which plays a role in selective autophagy and recognition of autophagic cargo, at the late phase also indicates that autophagic flux is not only initiated but also maintained by selective mechanisms targeting cellular protein and organelle homeostasis. Although *SQSTM1* expression showed a repressed profile in the early and middle phases, the ongoing change at the late phase indicates that the cellular recycling processes through autophagy are dynamically regulated.

Moreover, changes in the *TFEB* and *LAMP1* genes that play a role in the transcriptional and lysosomal regulation of autophagy around the 48th hour also suggest that lysosomal functions and autophagy–lysosome axis play a significant role in the cellular adaptation to TMZ for a long period of time. In particular, an increase in *TFEB* expression can be considered a finding confirming that autophagy and lysosome biogenesis are coordinately activated.

Overall, these data indicate that the autophagy early phase was initiated as a response to TMZ application; however, the extension and selective autophagy steps became dominant in late phase and the cells used these processes for survival and adaptation under long-term stress.

Exposure to TMZ for an extended period significantly alters the expression levels of genes involved in lysosomal functions. Especially at the late time point (48 h), the expression profiles of the genes that play a role in lysosome biogenesis and autophagy–lysosome axis regulation have been identified as being susceptible to TMZ treatment.

Changes in the expression level of the *LAMP1* gene, which is responsible for the protection of the lysosomal structure and functional integrity, at the middle and late phase, indicate that lysosomes are actively reconstituted during the later stages of the autophagic process. This situation shows that autophagy is not only limited to the formation of the autophagosome but also that lysosomal capacity and functions are reshaped as a part of the adaptive response to TMZ.

Moreover, the fact that the expression of *TFEB* gene, which plays a key role in lysosome biogenesis and transcriptional regulation of genes related to autophagy, shows a marked increase, especially at the late phase, indicates that long-term exposure to TMZ activates the autophagy–lysosome system. *TFEB* mediated this regulation; the cells were thought to be targeting the breakdown of autophagic cargo and the maintenance of cellular homeostasis through an increase in lysosomal activity.

Overall, these findings reveal that prolonged TMZ exposure causes lysosomal functions in T98G cells to be directly or indirectly affected, through which the later stages of the autophagy flux are reprogrammed, and lysosomes have become an important component of the cellular adaptive response to TMZ.

Following prolonged treatment with TMZ, significant changes have been observed in expression levels of genes involved in the regulation of lysosomal functions. In particular, at a late time point (48 h), it was found that the expression profiles of genes involved in lysosomal biogenesis and regulation of the autophagy–lysosome axis are sensitive to TMZ exposure.

Changes in the expression levels of the *LAMP1* gene, which are responsible for preserving the structure and functional integrity of lysosomes, in the middle and late phases suggest that lysosomes are actively rearranged through the later stages of autophagic processes. This indicates that autophagy is not only about the formation of autophagosomes, but also that lysosomal capacity and functions are reshaped as an adaptive response to TMZ.

Moreover, the increase in the expression of the *TFEB* gene, which plays a key role in transcriptional regulation of genes related to lysosome biogenesis and autophagy, was observed especially at the late stage. This points to the fact that continuous exposure to TMZ results in a holistic activation of the autophagy–lysosome system. This *TFEB*-mediated regulation implies that cells, through increased lysosomal activity, are aiming at efficient degradation of autophagic cargo and thus maintenance of cellular homeostasis.

These findings overall indicate that prolonged TMZ exposure in T98G cells led to remodeling of the late stages of autophagy by affecting lysosomal functions either directly or indirectly. As a result, the lysosomes become an integral component of the adaptive response to TMZ at the cellular level.

**Figure 7:**
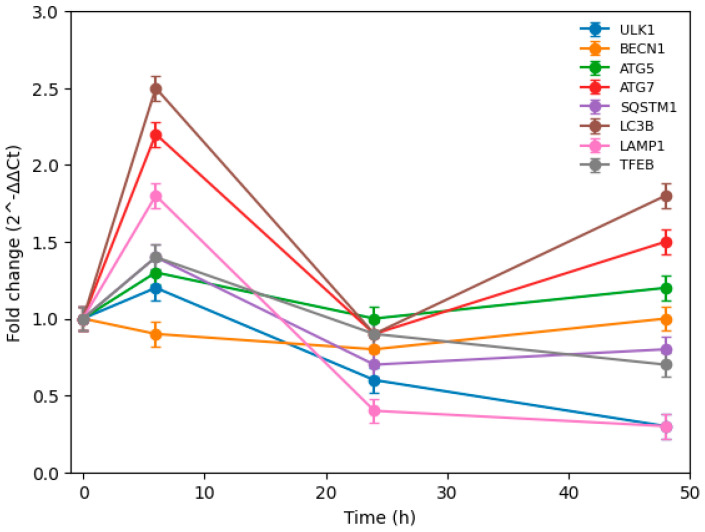
Time-dependent expression profile of autophagy-related genes in U87 glioblastoma cells following temozolomide (TMZ) treatment.

Relative mRNA expression levels of *ULK1*, *BECN1*, *ATG5*, *ATG7*, *SQSTM1* (*p62*), *LC3B*, *LAMP1*, and *TFEB* were determined by RT-qPCR at 0 h, 6 h, 24 h, and 48 h after treatment with 100 µM TMZ (Figure 7). Gene expression levels were normalized to *GAPDH* and calculated using the 2^−ΔΔCt^ method. Data are presented as fold change relative to the 0 h vehicle control and expressed as mean ± SEM of at least three independent experiments.

During the nucleation phase of autophagy in U87 cells, the expression profile of *BECN1*, one of the key regulators of autophagic nucleation, did not show a marked induction in the early phase following TMZ exposure. At 6 h, *BECN1* expression remained slightly below basal levels, and this modest or repressed pattern persisted up to 24 h. This suggests that although an early initiation signal may be triggered, the nucleation step is either tightly regulated or not strongly sustained in U87 cells during the early stress response.

At the later time point (48 h), *BECN1* expression returned approximately to basal levels, while *ULK1* expression remained suppressed compared to control. This pattern indicates that, unlike a fully coordinated adaptive program, the initiation and nucleation phases in U87 cells are not robustly maintained over prolonged TMZ exposure.

RT-qPCR analysis revealed clear time-dependent alterations in autophagy-related gene expression in U87 glioblastoma cells treated with TMZ. Relative expression levels were calculated using the 2^−ΔΔCt^ method normalized to *GAPDH* and are presented as fold change relative to the 0 h vehicle control. Data represent mean ± SEM of at least three independent experiments.

Following TMZ treatment, a pronounced early induction was observed at 6 h in several genes associated with autophagosome elongation and processing. In particular, *ATG7* and *LC3B* showed strong upregulation in the early phase, indicating rapid activation of autophagic machinery. However, at 24 h, the expression levels of most genes—including *ULK1*, *ATG7*, *SQSTM1*, and *LAMP1*—declined toward or below basal levels. This transient pattern suggests that the early autophagic response in U87 cells is not sustained over time.

At 48 h, partial reactivation of elongation-related genes such as *ATG5*, *ATG7*, and *LC3B* was observed. However, genes associated with lysosomal function and autophagy–lysosome axis regulation exhibited a different pattern. *LAMP1* expression remained markedly suppressed, and *TFEB* expression decreased below basal levels, indicating limited lysosomal adaptation during prolonged exposure.

In parallel, *SQSTM1* (*p62*) displayed a dynamic pattern characterized by early induction followed by suppression and only modest recovery at 48 h. This fluctuation may reflect dysregulated or incomplete autophagic flux rather than sustained cytoprotective autophagy.

Moreover, the marked reduction in *LAMP1* expression at middle and late phases suggests that lysosomal structural and functional integrity may be compromised in U87 cells under prolonged TMZ stress. In contrast to a coordinated lysosomal biogenesis response, the limited induction of *TFEB* indicates that transcriptional activation of the autophagy–lysosome system is not robustly sustained.

Overall, these findings indicate that although an early autophagic response is initiated in U87 cells upon TMZ exposure, the subsequent elongation and lysosomal regulatory phases do not appear to be efficiently maintained. Rather than reflecting a structured adaptive mechanism, the temporal expression pattern suggests a transient and possibly dysregulated autophagic response.

Prolonged TMZ exposure therefore does not seem to promote a fully integrated autophagy–lysosome adaptive program in U87 cells. Instead, the lack of sustained lysosomal activation and the suppression of key regulatory genes at later time points may contribute to the higher TMZ sensitivity observed in this cell line.

## 4. Discussion

Time-dependent and phase-specific regulation of autophagy observed in glioblastoma cells after TMZ treatment reveals the complex interplay between cellular stress response and chemoresistance mechanisms [37]. The detection of time-dependent significant changes in the expression of genes representing the initiation, elongation and lysosomal function steps of autophagy indicates that the autophagic machinery is dynamically reprogrammed in response to TMZ [38]. These results also suggest that autophagy is not a monotonously increasing or decreasing process but rather a multi-stage adaptation response progressing through phase-specific transcriptional regulations [39,40].

There are contradictory results reported in the literature regarding the role of autophagy in glioblastoma’s response to TMZ; some studies have suggested that autophagy supports cell survival, while others are in favor of autophagy inhibition to increase treatment efficacy [5,41]. However, the data obtained in this study indicate that the multifaceted and phase-specific nature of autophagy can explain these contradictions. Different stages of autophagy can have different effects on cell survival and drug sensitivity [10,42].

Induction of autophagy genes, especially at the very early phase, may facilitate a cytoprotective mechanism by which cells recycle their components to the stress caused by TMZ and thus adapt. Later, lysosomal function-related processes being highlighted may contribute to long-term resistance [24]. This indicates that autophagy plays different biological roles during the early and late phases.

The accumulation of autophagosomes in the process of autophagosome–lysosome fusion (i.e., the autophagic flux) being disrupted and the decreased expression of lysosomal proteins lead to the notion that the changes at the terminal stages of autophagy flux play a key role in the resistance to TMZ [43]. Therefore, not only the increase in autophagosome formation but the overall regulation of autophagic flux seems to be very critical in terms of chemoresistance. In fact, the accumulation of autophagosomes can lead to autophagic cell death under certain conditions; thus, the delicate balance between the protective and harmful effects of autophagy has been demonstrated [15].

The effects of TMZ treatment on LC3-II levels which were enhanced in the presence of bafilomycin A1 thus do not support the notion of suppressed autophagy but rather suggest a compensatory response developed against a heightened autophagic flux [20]. The dynamic equilibrium between autophagosome formation and lysosomal degradation explains why autophagy as a therapeutic target in glioblastoma leads to complex and contradictory results [4,5].

This complexity is further supported by the fact that different cellular responses are observed when autophagy modulators are used. For example, although drugs such as tamoxifen and rapamycin can induce autophagy, depending on the dose and cellular context, a transient increase in autophagy, cytoprotection or cell death can occur [25]. Likewise, autophagy inhibitors such as chloroquine and bafilomycin A1, by targeting different steps of autophagic flux, can modify the effectiveness of TMZ in a variable manner [44].

While primary glioblastoma exhibits pronounced intratumoral heterogeneity in vivo, our findings in homogeneous in vitro models (T98G and U87) suggest that the autophagic response itself is highly context-dependent and varies significantly with the intrinsic genetic background of the cells. This study reveals that autophagy in glioblastoma cells exposed to TMZ is not a static process but rather a time-dependent and multi-phase adaptation program. Autophagy in glioblastoma is dominantly a cytoprotective mechanism and this feature constitutes a major component of resistance against conventional therapies [9,13]. Therefore, phase-specific and timing-based targeting of autophagy may be a key strategy for the overcoming of TMZ resistance.

**Figure 8:**
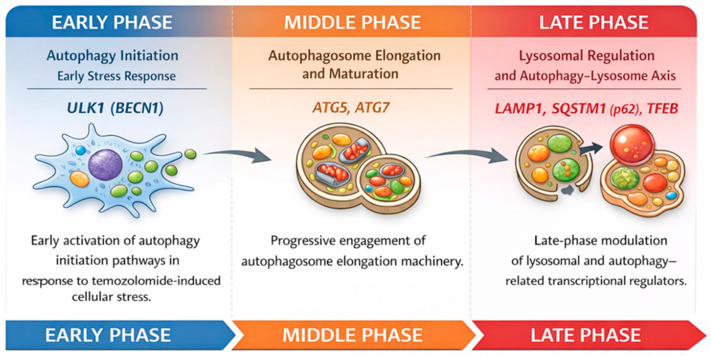
Schematic representation of the time-dependent and phase-specific regulation of autophagy-related genes in T98G glioblastoma cells following temozolomide treatment.

We propose a conceptual model summarizing phase-specific regulation of autophagy in glioblastoma cells under temozolomide treatment based on our time-dependent gene expression data (Figure 8). This model holistically illustrates early-phase autophagy induction, mid-phase autophagosome accumulation, and late-phase lysosomal dysfunction.

Early-phase responses (6 h) are characterized by modulation of autophagy initiation-related genes, predominantly *ULK1*. During the middle phase (24 h), genes involved in autophagosome elongation, including *ATG5* and *ATG7*, become prominent, indicating sustained autophagic activity. Prolonged exposure (48 h) is associated with regulation of lysosomal and transcriptional regulators such as *LAMP1*, *SQSTM1* (*p62*), and *TFEB*, suggesting late-stage adaptation of the autophagy–lysosome axis. This model is based on transcriptional profiling and does not directly assess autophagic flux or functional outcomes.

In the present research, temozolomide (TMZ) application has been shown to time-dependently and phase-specifically reprogram the expression of autophagy-related genes in T98G glioblastoma cells. The findings obtained indicate that, in response to TMZ, autophagy is not a unidirectional or static process; on the contrary, it represents a dynamic adaptation response during which different gene groups dominate at the early, middle and late stages of the process.

In this study, the time-dependent effects of temozolomide (TMZ) on autophagy-related gene expression were comparatively investigated in two glioblastoma cell lines with different sensitivity profiles (T98G and U87). The findings indicate that while TMZ triggers an autophagic response in both cell lines, the coordination, continuity, and lysosomal integration of this response differ significantly. Dose–response analyses revealed that the U87 cell line exhibits higher sensitivity to TMZ compared to T98G cells. Notably, the marked decrease in IC50 values in U87 cells, especially upon prolonged exposure, supports a time-dependent increase in cytotoxic sensitivity. In contrast, T98G cells maintained partial viability even at high doses and prolonged exposures, suggesting the involvement of robust adaptive cellular mechanisms.

At the transcriptional level, TMZ administration led to the activation of autophagy-initiating genes in the early phase in both cell lines. However, at subsequent time points, the gene expression profiles diverged considerably. In T98G cells, autophagy-related gene expression demonstrated a temporally organized progression. Following early initiation signals, an increase in the elongation-associated genes *ATG5* and *ATG7* occurred, followed by upregulated *TFEB* and *LAMP1* expression in the late phase. This suggests a holistic activation of the autophagy–lysosome axis, integrating lysosomal capacity into the cellular adaptation process. Thus, the autophagic response in T98G cells appears to function as a cytoprotective and adaptive mechanism.

Conversely, U87 cells exhibited a more transient and dysregulated gene expression pattern. Although early marked increases were observed in ATG7 and LC3B, this response was suppressed by 24 h, and a fully coordinated lysosomal adaptation did not develop in the late phase. The significant decline in *LAMP1* expression during the middle and late phases, along with limited *TFEB* activation, indicates that lysosome biogenesis and autophagy–lysosome integration were not sustainable. This implies that despite early autophagy initiation in U87 cells, the process was not effectively maintained, and lysosomal functions were insufficiently supported. Efficient autophagic flux requires the coordinated action of both autophagosome formation and lysosomal degradation. In U87 cells, the suppression of lysosomal regulatory genes may have limited the adaptive potential of autophagy, culminating in enhanced cytotoxicity. Overall, while TMZ induces an autophagic response in glioblastoma cells, the functional outcome is highly cell-line-dependent. In relatively resistant T98G cells, autophagy matures over time into an adaptive program, whereas in more sensitive U87 cells, early activation cannot be sustained, resulting in inadequate lysosomal compensation.

We acknowledge certain limitations in the present study. Most notably, autophagy was assessed primarily at the transcriptional level. Given that mRNA expression does not always strictly correlate with protein levels to confer biological activity, the lack of direct protein-level evaluations (such as LC3B-II accumulation or p62 degradation assays) and functional autophagic flux measurements is a limitation. Future studies incorporating protein-level validations and functional assays are warranted to fully confirm the biological and translational impact of these transcriptional dynamic changes.

## Figures and Tables

**Table 1:** Primer sequences for RT-qPCR analysis.

Gene	Forward Primer (5′–3′)	Reverse Primer (5′–3′)	Reference
*ULK1*	AGCAGTTGCTTCTGTTGCTC	TCTGCTGATGGTGATGTTGG	[28]
*TFEB*	CAGTGGAGCAGAGAGACTTTG	CTCTTCTGGTAGCTGCTGGT	[29]
*WIPI1*	GCTGCTGTTGCTACTGTTGA	AGGTAGCTGCTGTTGATGGT	[30]
*NBR1*	TGGACAGCTTCAGTCTTCGT	CTGTAGGTGGAGGCTTTGGT	[31]
*ATG5*	CAGTTTGGCACACACTTGTG	GTCTGTGATGGGTTGTTGCT	[32]
*ATG7*	ACACCAAGAGGAGCTGTTGA	TGTGCTGTTGCTGTAGGTGT	[33]
*LC3B*	GAGAAGCAGCTTCCTGTTCT	CTCCTGGGAGGCATAGACAT	[34]
*FOXO3*	GCGTGCCCTACTTCAAGGATA	TGGGAGTTCCTTCATTCTGGT	[35]
*GAPDH*	GAAGGTGAAGGTCGGAGTC	GAAGATGGTGATGGGATTTC	[36]

**Table 2:** Cell viability of T98G and U87 glioblastoma following time- and dose-dependent temozolomide treatment.

Time (h)	Condition	TMZ (µM)	T98G Mean Viability (%)	U87 Mean Viability (%)
6	Vehicle (DMSO)	0	98.13	98.13
6	TMZ	50	90.45	89.50
6	TMZ	100	77.01	72.41
6	TMZ	200	45.03	28.54
24	Vehicle (DMSO)	0	97.92	97.92
24	TMZ	50	81.77	79.95
24	TMZ	100	63.11	55.73
24	TMZ	200	32.96	12.85
48	Vehicle (DMSO)	0	99.62	99.62
48	TMZ	50	77.74	75.51
48	TMZ	100	58.82	50.58
48	TMZ	200	27.69	0.00 *

*: Below the limit of detection.

## Data Availability

The original contributions presented in this study are included in the article. Further inquiries can be directed to the corresponding author.

## Abbreviations

The following abbreviations are used in this manuscript:

ATG	Autophagy-related gene
BECN1	Beclin 1
CQ	Chloroquine
DMEM	Dulbecco’s Modified Eagle Medium
DMSO	Dimethyl sulfoxide
EGFR	Epidermal growth factor receptor
GBM	Glioblastoma multiforme
LC3	Microtubule-associated protein 1 light chain 3
LC3-II	Lipidated form of LC3
LAMP1	Lysosomal-associated membrane protein 1
LAMP2	Lysosomal-associated membrane protein 2
MGMT	O6-methylguanine-DNA methyltransferase
mTOR	Mechanistic target of rapamycin
PI3K	Phosphoinositide 3-kinase
PTEN	Phosphatase and tensin homolog
RT-qPCR	Reverse transcription quantitative polymerase chain reaction
SEM	Standard error of the mean
SQSTM1/p62	Sequestosome 1
TMZ	Temozolomide
ULK1	Unc-51 like autophagy activating kinase 1
ULK2	Unc-51 like autophagy activating kinase 2

## References

[1] Würstle S., Schneider F., Ringel F., Gempt J., Lämmer F., Delbridge C., Wu W., Schlegel J. (2017). Temozolomide induces autophagy in primary and established glioblastoma cells in an EGFR independent manner. Oncol. Lett..

[2] Hekmatshoar Y., Gürel A.K. (2023). Entegre miRNA/mRNA düzenleyici ağ analizi ile Glioblastomda temozolomid direnç faktörlerinin belirlenmesi. Ege Tıp Bilim. Derg..

[3] Sencar L., Yılmaz D., Göktürk D., Polat S., Çoşkun G., Şaker D., Sapmaz T., Kara S., Çelenk A., Polat S. (2021). Effects of cyclopamine and temozolomide combined treatment on miR-20a expression in glioblastoma cell line (U87). Çukurova Med. J..

[4] Yan Y., Xu Z., Dai S., Qian L., Sun L., Gong Z. (2016). Targeting autophagy to sensitive glioma to temozolomide treatment. J. Exp. Clin. Cancer Res..

[5] Jiapaer S., Furuta T., Tanaka S., Kitabayashi T., Nakada M. (2018). Potential Strategies Overcoming the Temozolomide Resistance for Glioblastoma. Neurol. Med.-Chir..

[6] Wang L., Shang Z., Zhou Y., Hu X., Chen Y., Fan Y., Wei X., Wu L., Liang Q., Zhang J. (2018). Autophagy mediates glucose starvation-induced glioblastoma cell quiescence and chemoresistance through coordinating cell metabolism, cell cycle, and survival. Cell Death Dis..

[7] He Y., Su J., Lan B., Gao Y., Zhao J. (2019). Targeting off-target effects: Endoplasmic reticulum stress and autophagy as effective strategies to enhance temozolomide treatment. DOAJ Dir. Open Access J..

[8] Buccarelli M., Marconi M., Pacioni S., Pascalis I.D., D’Alessandris Q.G., Martini M., Ascione B., Malorni W., Larocca L.M., Pallini R. (2018). Inhibition of autophagy increases susceptibility of glioblastoma stem cells to temozolomide by igniting ferroptosis. Cell Death Dis..

[9] Jandrey E.H.F., Bezerra M., Inoue L.T., Furnari F.B., Camargo A.A., Costa É.T. (2021). A Key Pathway to Cancer Resilience: The Role of Autophagy in Glioblastomas. Front. Oncol..

[10] Zhao Z., Liu M., Long W., Yuan J., Li H., Zhang C., Tang G., Jiang W., Yuan X., Wu M. (2021). Knockdown lncRNA CRNDE enhances temozolomide chemosensitivity by regulating autophagy in glioblastoma. Cancer Cell Int..

[11] Feng J., Zhang Y., Ren X., Li D., Fu H., Liu C., Zhou W., Liu Q., Liu Q., Wu M. (2020). Leucine-rich repeat containing 4 act as an autophagy inhibitor that restores sensitivity of glioblastoma to temozolomide. Oncogene.

[12] Huang H., Song J., Liu Z., Li P., Xu G. (2017). Autophagy activation promotes bevacizumab resistance in glioblastoma by suppressing Akt/mTOR signaling pathway. Oncol. Lett..

[13] Qin X., Xu M., Zhang P., Liu L., Meng X., Dong L. (2020). Therapeutic Potential of Autophagy in Glioblastoma Treatment with Phosphoinositide 3-Kinase/Protein Kinase B/Mammalian Target of Rapamycin Signaling Pathway Inhibitors. Front. Oncol..

[14] The Scientific Committee (2023). Proceedings of the 68th Congress of the Italian Embryological Group-Italian Society of Development and Cell Biology (GEI-SIBSC)—Oliveri, 5–8 June 2023. Eur. J. Histochem..

[15] Wan S., Zhang G., Liu R., Abbas M.N., Cui H. (2023). Pyroptosis, ferroptosis, and autophagy cross-talk in glioblastoma opens up new avenues for glioblastoma treatment. Cell Commun. Signal..

[16] Pu J., Yuan K., Tao J., Qin Y., Li Y., Fu J., Zhong L., Zhou H., Tang Z., Li L. (2025). Glioblastoma multiforme: An updated overview of temozolomide resistance mechanisms and strategies to overcome resistance. Discov. Oncol..

[17] Wen Z., Zeng W., Chen Y., He L., Wang J., Cheng Q., Yu J., Zhou H., Liu Z., Xiao J. (2019). Knockdown *ATG4C* inhibits gliomas progression and promotes temozolomide chemosensitivity by suppressing autophagic flux. J. Exp. Clin. Cancer Res..

[18] Teraiya M., Perreault H., Chen V. (2023). An overview of glioblastoma multiforme and temozolomide resistance: Can LC-MS-based proteomics reveal the fundamental mechanism of temozolomide resistance?. Front. Oncol..

[19] Poon W.S., Li Y., Anna C.H.Y., Stephanie N.C.P., Herbert L.H.F., Danny C.T.M., Wong G.K.C. (2019). A multifaceted review of temozolomide resistance mechanisms in glioblastoma beyond O-6-methylguanine-DNA methyltransferase. Glioma.

[20] Kriel J., Müller-Nedebock K.K., Maarman G.J., Mbizana S., Ojuka E.O., Klumperman B., Loos B. (2018). Coordinated autophagy modulation overcomes glioblastoma chemoresistance through disruption of mitochondrial bioenergetics. Sci. Rep..

[21] Singh N., Miner A., Hennis L., Mittal S. (2020). Mechanisms of temozolomide resistance in glioblastoma—A comprehensive review. Cancer Drug Resist..

[22] Smerdi D., Moutafi M., Kotsantis I., Stavrinou L.C., Psyrri A. (2024). Overcoming Resistance to Temozolomide in Glioblastoma: A Scoping Review of Preclinical and Clinical Data. Life.

[23] Hu Z., Mi Y., Qian H., Guo N., Yan A., Zhang Y., Gao X. (2020). A Potential Mechanism of Temozolomide Resistance in Glioma–Ferroptosis. Front. Oncol..

[24] Rahman M.A., Engelsen A.S.T., Sarowar S., Bindesbøll C., Birkeland E., Goplen D., Lotsberg M.L., Knappskog S., Simonsen A., Chekenya M. (2022). Bortezomib abrogates temozolomide-induced autophagic flux through an ATG5 dependent pathway. Front. Cell Dev. Biol..

[25] Khan I., Baig M.H., Mahfooz S., Rahim M., Karaçam B., Elbasan E.B., Ulasov I.V., Dong J.-J., Hatiboğlu M.A. (2021). Deciphering the Role of Autophagy in Treatment of Resistance Mechanisms in Glioblastoma. Int. J. Mol. Sci..

[26] Li H., Wu Y., Chen Y., Lv J., Qu C., Mei T., Zheng Y., Ye C., Li F., Ge S.S. (2025). Overcoming temozolomide resistance in glioma: Recent advances and mechanistic insights. Acta Neuropathol. Commun..

[27] Korkmaz M., Çelik B., ERSÖZ E. (2020). Investigation of the therapy potential of borax pentahydrate in glioblastoma multiforme cell line. J. Boron.

[28] Egan D.F., Shackelford D.B., Mihaylova M.M., Gelino S., Kohnz R.A., Mair W., Vasquez D.S., Joshi A., Gwinn D.M., Taylor R. (2011). Phosphorylation of ULK1 (hATG1) by AMP-Activated Protein Kinase Connects Energy Sensing to Mitophagy. Science.

[29] Settembre C., Di Malta C., Polito V.A., Garcia Arencibia M., Vetrini F., Erdin S., Erdin S.U., Huynh T., Medina D., Colella P. (2011). TFEB Links Autophagy to Lysosomal Biogenesis. Science.

[30] Polson H.E.J., de Lartigue J., Rigden D.J., Reedijk M., Urbé S., Clague M.J., Tooze S.A. (2010). Mammalian Atg18 (WIPI) Proteins Associate with Autophagosome Membranes. Nat. Cell Biol..

[31] Kirkin V., Lamark T., Sou Y.S., Bjørkøy G., Nunn J.L., Bruun J.A., Shvets E., McEwan D.G., Clausen T.H., Wild P. (2009). A Role for NBR1 in Autophagosomal Degradation of Ubiquitinated Substrates. Mol. Cell.

[32] Mizushima N., Levine B., Cuervo A.M., Klionsky D.J. (2008). Autophagy Fights Disease through Cellular Self-Digestion. Nature.

[33] Komatsu M., Waguri S., Ueno T., Iwata J., Murata S., Tanida I., Ezaki J., Mizushima N., Ohsumi Y., Uchiyama Y. (2005). Impairment of Starvation-Induced and Constitutive Autophagy in Atg7-Deficient Mice. J. Cell Biol..

[34] Kabeya Y., Mizushima N., Ueno T., Yamamoto A., Kirisako T., Noda T., Kominami E., Ohsumi Y., Yoshimori T. (2000). LC3, a Mammalian Homologue of Yeast Apg8p, Is Localized in Autophagosome Membranes after Processing. EMBO J..

[35] Warr M.R., Binnewies M., Flach J., Reynaud D., Garg T., Malhotra R., Debnath J., Passegué E. (2013). FOXO3A Directs a Protective Autophagy Program in Haematopoietic Stem Cells. Nature.

[36] Barber R.D., Harmer D.W., Coleman R.A., Clark B.J. (2005). GAPDH as a Housekeeping Gene: Analysis of GAPDH mRNA Expression in a Panel of 72 Human Tissues. Genome Res..

[37] Chu C., Yang M., Chou C., Huang W., Hsiao B., Wang Y., Chiou S., Loh J., Hong Y. (2018). GSK3β mediated Ser156 phosphorylation modulates a BH3 like domain in BCL2L12 during TMZ induced apoptosis and autophagy in glioma cells. Int. J. Mol. Med..

[38] Yun E.-J., Kim S., Hsieh J., Baek S.T. (2020). Wnt/β-catenin signaling pathway induces autophagy-mediated temozolomide-resistance in human glioblastoma. Cell Death Dis..

[39] Abbas S., Singh S.K., Saxena A.K., Tiwari S., Sharma L.K., Tiwari M. (2020). Role of autophagy in regulation of glioma stem cells population during therapeutic stress. J. Stem Cells Regen. Med..

[40] Chaicharoenaudomrung N., Kunhorm P., Promjantuek W., Rujanapun N., Heebkaew N., Soraksa N., Noisa P. (2019). Transcriptomic Profiling of 3D Glioblastoma Tumoroids for the Identification of Mechanisms Involved in Anticancer Drug Resistance. In Vivo.

[41] Xu S., Luo C., Chen D., Tang L., Chen L., Liu Z. (2022). Whole transcriptome and proteome analyses identify potential targets and mechanisms underlying tumor treating fields against glioblastoma. Cell Death Dis..

[42] Wang Y., Zhang B., Wang J., Wu H., Xu S., Zhang J., Wang L. (2021). Discovery of LAMP-2A as potential biomarkers for glioblastoma development by modulating apoptosis through N-CoR degradation. Cell Commun. Signal..

[43] Shojaei S., Basso J., Amereh M., Alizadeh J., Dehesh T., Rosa S.C.d.S., Clark C., Hasan M., Tomczyk M.M., Cole L. (2022). A multi-omics analysis of glioma chemoresistance using a hybrid microphysiological model of glioblastoma. bioRxiv.

[44] Cristofori A.D., Ferrero S., Bertolini I., Gaudioso G., Russo M.V., Berno V., Vanini M., Locatelli M., Zavanone M., Rampini P. (2015). The vacuolar H+ ATPase is a novel therapeutic target for glioblastoma. Oncotarget.

